# Variability in article processing charges of open-access publishing across medical specialties

**DOI:** 10.1371/journal.pone.0320684

**Published:** 2025-07-30

**Authors:** Emily G. Tocco, Mackenzie M. Mayhew, Margaret G. Mercante, Nidhi Kuchimanchi, Mohamad El Moheb, Chengli Shen, Allan Tsung, Russell G. Witt

**Affiliations:** 1 Department of Surgery, University of Virginia, Charlottesville, Virginia, United States of America; Max Planck Institute for Solid State Research, GERMANY

## Abstract

**Background:**

Open-access (OA) journals provide public access to research but often impose a financial burden on researchers through article processing charges (APCs). The connection between APCs and indicators of journal prestige, such as journal impact factor (JIF), and their variation across medical specialties remains unclear. This study investigates how publication costs relate to journal prestige across diverse medical fields.

**Methods:**

Data from 1,117 hybrid and OA journals across 29 medical specialties were compiled from Journal Citation Reports and journal websites. Pearson correlation coefficients between APCs and journal characteristics (JIF, journal citation indicator, citation counts, and percentage of OA publications) were calculated. Linear regression was used to assess the impact of these journal characteristics on APC variance. Bartlett’s test for homogeneity of variances was performed to evaluate differences in APC variance across specialties and publishing models. Journal counts by country of origin and corresponding median APCs were also analyzed.

**Results:**

Significant variation in APCs was observed across specialties, with hematology/oncology journals having the highest median APC ($4,690) and primary care journals the lowest ($2,690). Hybrid journals had higher median APCs than OA journals ($4,248 vs. $2,909, p < 0.001). JIF and the proportion of OA publications accounted for only 14.1% of the APC variance in Q1 journals. Weak positive correlations were found between APC and both JIF (r = 0.38) and citation counts (r = 0.38), and a weak negative correlation between APC and the proportion of OA publications (r = −0.28).

**Conclusions:**

Across all medical specialties, hybrid journals have higher APCs than fully open-access journals. Although APCs vary within specialties, differences across fields are less pronounced. The weak correlations between APC and journal prestige metrics suggest that factors such as JIF, citation counts, and the proportion of OA publications account for only a small fraction of APC variability.

## Introduction

Open-access (OA) journals increase the accessibility of research to a global audience by eliminating reader paywalls [1]. The unrestricted accessibility of OA articles is associated with higher citation rates compared to non-OA articles [2], and research published in OA journals often receive greater media attention, [3] providing strong incentives for researchers to prioritize open-access dissemination. Many journals are moving towards full open access through a model known as Transformative Journals (TJs), which are hybrid journals committed to becoming fully OA over time by increasing the proportion of OA articles published [4]. These developments reflect a broader shift in the publishing landscape toward greater transparency in the dissemination of scientific knowledge.

Although the digitization of published articles makes scientific knowledge more accessible globally, it resulted in a loss of income for publishers due to reductions in reprint and journal subscription costs. A shift from charging readers to charging authors, in the form of article processing charges (APCs), was implemented to compensate for this income loss [5]. While these values range based on journal, APCs may pose a challenge for researchers with limited funding, whereas those with greater resources may prioritize journal prestige over cost, contributing to inequities in who is able to publish in high-visibility open access journals [6–10]. Additionally, the factors driving APC variation remain unclear, complicating efforts to ensure that research funding is allocated efficiently toward high-quality and impactful publication.

Understanding the factors influencing APC variation is critical, as these charges significantly impact the accessibility of open-access publishing. Previous research has provided insights into publishing costs within individual medical specialties [1,11] evaluating characteristics such as journal prestige, geographic location, publication frequency, and publisher within specific medical fields [7,12]. However, there has been limited investigation into the broader determinants of APC variation across wide range of medical specialties. This study aims to address these gaps by investigating the variation in APCs across 29 medical specialties, assessing the influence of journal prestige, publishing models, and countries of origin.

## Methods

### Journal and specialty selection

In this cross-sectional study, data on both hybrid and open-access journals in 29 different medical specialties were compiled from Journal Citation Reports (JCR) and journal websites. Data collection occurred between June and July 2024 and was conducted by three investigators, each assigned a subset of specialties: Investigator A reviewed journals in Allergy through Family Medicine, Investigator B covered Internal Medicine through Orthopedics/Sports Medicine, and Investigator C handled Oncology through Urology. Each journal was assigned to the same medical specialty as designated in the JCR database. The following specialties were analyzed: Allergy, Anesthesiology, Cardiology, Clinical Neurology, Critical Care Medicine, Dermatology, Emergency Medicine, Endocrinology & Metabolism, Gastroenterology & Hepatology, Geriatrics & Gerontology, Hematology, Immunology, Infectious Diseases, General/Internal Medicine, Obstetrics & Gynecology, Oncology, Ophthalmology, Orthopedics, Otorhinolaryngology, Pathology, Pediatrics, Primary Health Care, Psychiatry, Psychology, clinical, Radiology, Rehabilitation, Rheumatology, Surgery, Urology & Nephrology. Journal country of origin was determined using publisher information listed in JCR, which reflects address and affiliation details submitted by the journal or publisher. Journal country income was determined based on World Bank classifications [13]. Journals missing information on APC or publishing model type were excluded. Journals with an APC of $0 or diamond open access were also excluded to focus on variability of fee-based open access journals. APC data came directly from the official website of each journal. To maintain uniformity, the APC for an “Original Article” was the APC collected for each journal. For journals listing APCs in currencies other than US dollars, values were converted using the Google currency conversion tool which obtains exchange rates from Morningstar.

### Measures of journal prestige

Journal prestige was assessed using established bibliometric indicators available from the Journal JCR database. These included the Journal Impact Factor (JIF), a widely used metric representing the average number of citations received by articles published in a journal during a specific period, and the Journal Citation Indicator (JCI), which reflects normalized citation impact across disciplines. Citation counts, which measure the total number of citations received by a journal, were also used as an additional indicator of journal influence. For this study, the JIF per journal was collected from the year 2023. To maintain consistency and ensure data quality, only journals in the top quartile of JCR rankings were included, as this group is assumed to exhibit greater prestige, less variability in quality, and a lower likelihood of including predatory journals. These measures were analyzed to determine their relationship with APCs and their role in explaining APC variation across specialties, publishing models, and countries of origin.

### Statistics

Measures of central tendency for APCs and Spearman correlation coefficients between APCs and various journal characteristics, including JIF, JCI, citation counts, and percentage of open access papers within the journal, were assessed for each specialty. Two multivariable linear regression models were constructed to evaluate predictors of APCs. The first model included main effects for specialty, 2023 JIF, and the percentage of open access publications within a journal. The second model added an interaction term between specialty and 2023 JIF to assess whether the association between JIF and APC varied by specialty.

To determine if variances in APC were consistent across specialties and publishing models, we performed Bartlett’s test for homogeneity of variances. Bartlett’s K-squared values, degrees of freedom, and p-values were calculated to identify significant differences in APC variances by specialty and model. Journal counts by country of origin, along with the corresponding median APC for each country, were also analyzed. All included journals had complete data for the variables analyzed. Country of origin data were missing for only 5 journals, which were retained in the overall analysis but excluded from any country-level comparisons. This study adheres to the Strengthening the Reporting of Observational Studies in Epidemiology (STROBE) guidelines for cross-sectional studies.

## Results

**Fig 1:**
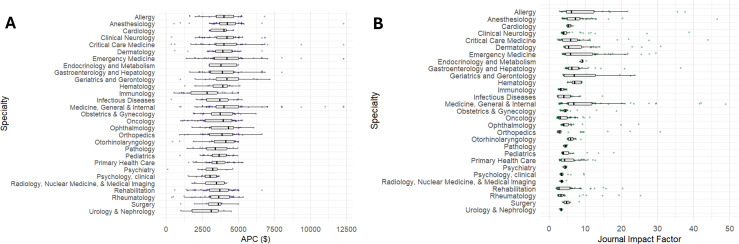
A) Article processing charges by specialty. B) Journal Impact Factor (from the year 2023) by specialty.

Of the 1,117 journals compiled from the Journal Citations Report, 1,007 journals were included. 80 journals were excluded due to lack of online transparency about either the type of publishing model the journal uses or the APC, and 30 were excluded for having an APC of $0. Of those excluded for an APC of $0, 13 were diamond open access journals from 8 different specialties (S1 Table). We found a significant variance in mean APC prices between journal specialties (p < 0.001), with hematology journals having the highest mean APC ($4,621) and primary care journals the lowest ($2,565). Similarly, mean JIF varied significantly across specialties (p < 0.001), with oncology journals having the highest mean JIF (18.3) and rehabilitation journals the lowest (3.0). Figs 1A and 1B show the distribution of APCs and JIFs by specialty, respectively. Hybrid journals were found to have a significantly higher median APC than OA journals ($4,248 vs. $2,909, p < 0.001) (Table 1).

**Table 1:** Descriptive statistics of article processing charges by publishing mode.

Publishing Model	Number of Journals	Median ([IQR]) ($)
Hybrid	698	4190 (3562-4720)
Open Access	307	2940 (2357-3495)
Subscription	2	8245 (6222-10268)

**Fig 2:**
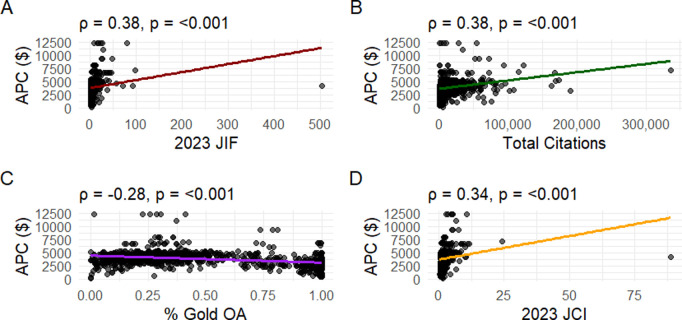
Spearman correlation of article processing charge (APC) and various journal metrics. The Spearman ρ and associated p-value are shown for each metric. Each point represents one journal. **A)** Correlation between APC and 2023 Journal Impact Factor (JIF). **B)** Correlation between APC and total citations. **C)** Correlation between APC and percent of gold open access (gold OA) articles within a journal. **D)** Correlation between APC and 2023 Journal Citation Indicator (JCI).

Weak positive correlations were observed between APC and JIF (r = 0.38, p < 0.001), citation counts (r = 0.38, p < 0.001), and JCI (r = 0.34, p < 0.001). In contrast, a weak negative correlation (r = −0.28, p < 0.001) was observed between APC and the proportion of open-access publications for each journal (Figs 2A-D).

Bartlett’s test is a statistical method used to assess whether there are significant differences in the variability of a given measure across multiple groups. In this study, it was used to evaluate whether the APC significantly differed across various medical specialties and publishing models. Bartlett’s K-squared value was 138.92 with 28 degrees of freedom for variance in APC between specialties (p<<0.001), while the Bartlett’s K-squared value for variance in APC across models was 45.61 with 2 degrees of freedom (p < 0.001), suggesting variance in APCs across both specialty and model (Table 2). These findings suggest that there is significant variation in APCs when grouped by specialties and by publishing models. Further, APC variability is not uniform and depends on these categorical factors. The highest standard deviations in APCs were observed in geriatrics and gerontology (2,146) and oncology (2,015), while the lowest standard deviations in APC were seen in clinical psychology (765) and pathology (746). When normalized using the coefficient of variation (CV), geriatrics and gerontology (CV = 0.48) and oncology (CV = 0.44) remained the most variable with respect to APC, while clinical psychology (CV = 0.22) and pathology (CV = 0.18) remained the least variable in APCs, reflecting more consistent pricing structures.

**Table 2:** Bartlett’s test for homogeneity of variances by specialty and model.

Factor	Bartlett’s K-squared	Degrees of Freedom (df)	p-value
Specialty	138.92	28	<0.001
Model	45.61	2	<0.001

**Fig 3:**
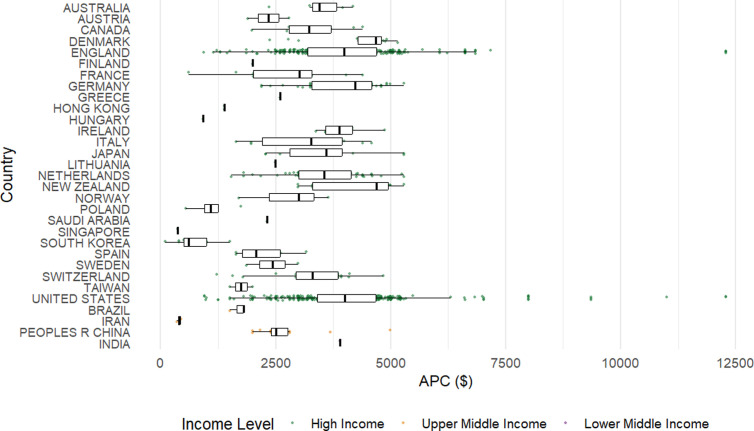
Article processing charge by country. Each point represents a journal, and the point colors denote the income level of the country of the journal according to the World Bank.

In both regression models, a higher percentage of open access publications was associated with significantly lower APCs with APCs decreasing by $79.40 in the main effects model (p < 0.001) and $75.90 in the interaction model (p < 0.001) for every 5% increase in gold open access content. In the interaction model, oncology was the only specialty with significantly higher APCs compared to the reference group of allergy with an increase of $2,709 (p = 0.033). JIF (p = 0.18) and its interaction with specialty (all p > 0.05) were not statistically significant. The main effects model explained 23.9% of the variance in APCs, while the interaction model explained 34.6%, indicating that inclusion of the interaction modestly improved explanatory power. Analysis of APC based on journal country revealed significantly higher mean APCs in several countries, including Denmark, New Zealand, England, Germany, Ireland, and the United States, as compared to Asian countries such as Taiwan, Hong Kong, South Korea, and Iran. New Zealand had the highest median APC at $4,690, while Singapore had the lowest median APC of $373 (Figure 3). The United States had the most journals (449) in the top JCR quartile across specialties, followed by England (299) and the Netherlands (54).

## Discussion

The results of our study highlight the complexity and variability of APCs across medical specialties and publishing models. While open-access publishing has increased access to scientific knowledge, the significant differences in APCs raise questions about the equity and transparency of the publishing ecosystem. Weak correlations between APCs and journal prestige metrics indicate that pricing is influenced by factors beyond traditional measures of journal quality. Furthermore, hybrid journals were consistently found to have higher APCs than fully open-access journals, and significant variance in APCs across specialties suggests that these costs are not uniformly applied. Importantly, the ability of publishers to set APCs without jeopardizing market share may contribute to hyperinflation [9]. These findings underscore the need for greater transparency in APC pricing.

The correlations between APC and measures of journal prestige were weak across medical specialties, indicating that these factors have minimal influence on determining APCs despite their potential to influence authors’ choice of journals for publication. This finding aligns with prior research showing minimal correlation between APCs and impact factors in open access surgery journals [1] and no association between high APCs and JIF or processing times in gastroenterology journals [12]. In contrast, a separate investigation of indexed ophthalmology journals in 2019 showed that impact factor, publication mode, and commercial publishers were positively correlated with APCs [14]. Another study found that while APCs did not correlate with the total number of open access and paid articles published by hybrid urology journals, there was a notable correlation between the total number these articles and CiteScore, a metric of the average number of yearly citations to published articles in a journal [15]. The relationship between journal prestige metrics and APCs appears to vary both within and across specialties, and our findings indicate that overall journal prestige has a limited impact on APC pricing.

Overall, our results found that hybrid journals charged higher APCs than fully open access journals. This is consistent with Gardner *et al*. (2021), who highlighted that hybrid OA oncology journals had an average higher APC than fully OA oncology journals. A study examining factors influencing APCs in open access and hybrid journals found that, for fully open access journals, APCs are mainly determined by the journal’s impact factor and the volume of articles published; in contrast, for hybrid journals, APCs are also influenced by the journal’s subscription fees [16]. In this way, hybrid journals have a unique advantage because they generate revenue from both subscription fees and APCs. Additionally, multivariable linear regression found that only 34.6% of the variation in APCs can be explained by specialty, JIF, and the percentage of OA Gold content. Notably, about 65% of the variation in journal prices remains unexplained, pointing to factors beyond journal prestige which are unknown to authors. Further research is necessary to identify these factors, particularly as publishers increasingly transition from subscription-based models to open access.

Although both journal specialty and publishing model showed statistically significant variances in APCs, the data suggest that APC variance is more strongly associated with journal specialty than publishing model. For instance, journals in specialties such as geriatrics and gerontology and oncology exhibited higher variances in APC than those observed across publishing models, with the exception of subscription-based journals, which showed a large variance in APCs.

Our findings demonstrate considerable variation in APCs across countries of different income classifications, including within high-income settings. Nearly all top-quartile journals are based in high-income countries with only one lower-middle income country and no low-income countries, highlighting a geographic concentration of prestige. Although the single journal from a lower-middle-income country reported a similar APC to the median of high-income countries, the spread among high-income countries was broad ($100 – $12,290). Some, like the United States and Denmark, had notably high APCs, while others such as Hungary, Poland, and Singapore, had much lower costs despite also being high income. Journals from upper-middle-income countries tended to have lower APCs with a median of $2,400. This disparity is further exacerbated by the tendency of hybrid journals to offer fewer APC waivers to authors from economically disadvantaged regions [17–19]. Abdel-Razig et al. underscored this disparity, showing that authors from lower- and middle-income countries face disproportionately higher relative costs of publishing, as observed in Health Professions Education journals [20]. Moreover, perceptions and attitudes toward APCs may vary depending on access to external funding and institutional support, of which researchers in lower-middle and lower-income countries have less access to further compounding academic inequity [8].

Although journal prestige is often cited to justify higher APCs, literature suggests that factors such as publisher size and market demand play a more significant role in determining these fees than traditional quality metrics like peer review rigor or dissemination scope [21]. This aligns with our analysis, which demonstrates weak correlations between APCs and journal prestige metrics, indicating that pricing may reflect market tolerance rather than actual publication costs [22]. To mitigate these costs, some institutions have established agreements with publishers that offer APC subsidies, while others engage in collective funding partnerships, allocate specific grant funds, or have societal partnerships for open-access publishing [7,23,24]. APC waivers are also sometimes provided by publishers, particularly to authors from lower-middle and lower-income countries, however, waiver policies may not be clearly disclosed on journal websites or may not be disclosed until after a manuscript is accepted, creating additional barriers for authors without substantial institutional or financial support [6].

Ultimately, our findings implicate broader concerns about equity, access, and sustainability in the biomedical sciences publishing landscape. As open-access models become increasingly more dominant, the financial burden of publishing risks shifting disproportionately onto researchers, institutions, and funders. Without greater transparency and regulation of APC pricing, the current system may reinforce or exacerbate existing disparities in who gets to publish their work widely. Understanding these patterns is essential not only for researchers navigating where to publish, but also for institutions, funders, and societies aiming to create a more inclusive and equitable landscape for scholarly communication.

Our study has several limitations. Data extractions were not independently cross-checked across investigators which may introduce the possibility of minor inconsistencies. Our analysis was restricted to journals in the top quartile of JCR rankings, which was intended to focus on high-prestige journals most likely to be targeted by authors. However, this design inherently introduces selection bias and limits generalizability to journals with lower impact factors or alternative publishing models. As a result, the observed relationships between APCs and journal characteristics may not reflect trends across the full spectrum of journals and may either underestimate or overestimate the variance explained by prestige. Additionally, data on submission or review charges were not explicitly collected. Some journals or publishers may offset lower APCs by imposing these additional fees not captured in our analysis. Journals that were diamond open access or with APCs of $0 were excluded to focus specifically on fee-based APC models. This may overlook potential differences in funding structures or cost transparency in journals that do not charge APCs. Future studies should include all JCR quartiles, journals with $0 APCs, diamond open access models, and submission or review charges to fully capture the range of publishing costs and models across all journals.

## Conclusion

Across all the medical specialties analyzed, authors face higher publication costs in hybrid journals compared to fully open access journals. While APCs show significant variation within specific medical fields, differences across fields as a whole are less pronounced. The association between journal prestige metrics and APCs was found to be weak. Additionally, journals of high prestige are concentrated in wealthier countries and tended to have higher APCs, though notably lower APCs were observed in many Asian countries with similar high-income backgrounds. Greater transparency in publishing costs is needed to ensure researcher time and funding is spent efficiently and directed toward high-quality, impactful dissemination of scientific work.

## Supporting information

S1 Table Diamond open access journals ranked within quartile 1. (XLSX)

S1 Data Quartile 1 journals and associated publishing costs. (XLSX)
